# Exploring the molecular mechanism of Xuebifang in the treatment of diabetic peripheral neuropathy based on bioinformatics and network pharmacology

**DOI:** 10.3389/fendo.2024.1275816

**Published:** 2024-02-08

**Authors:** Faquan Hu, Jiaran Lin, Liyuan Xiong, Zhengpin Li, Wen-ke Liu, Yu-jiao Zheng

**Affiliations:** ^1^ College of Traditional Chinese Medicine, Anhui University of Chinese Medicine, Hefei, China; ^2^ Affiliated Department of Endocrinology, Guang’anmen Hospital, China Academy of Chinese Medical Sciences, Beijing, China

**Keywords:** Xuebifang, diabetic peripheral neuropathy, bioinformatics, network pharmacology, traditional Chinese medicine

## Abstract

**Background:**

Xuebifang (XBF), a potent Chinese herbal formula, has been employed in managing diabetic peripheral neuropathy (DPN). Nevertheless, the precise mechanism of its action remains enigmatic.

**Purpose:**

The primary objective of this investigation is to employ a bioinformatics-driven approach combined with network pharmacology to comprehensively explore the therapeutic mechanism of XBF in the context of DPN.

**Study design and Methods:**

The active chemicals and their respective targets of XBF were sourced from the TCMSP and BATMAN databases. Differentially expressed genes (DEGs) related to DPN were obtained from the GEO database. The targets associated with DPN were compiled from the OMIM, GeneCards, and DrugBank databases. The analysis of GO, KEGG pathway enrichment, as well as immuno-infiltration analysis, was conducted using the R language. The investigation focused on the distribution of therapeutic targets of XBF within human organs or cells. Subsequently, molecular docking was employed to evaluate the interactions between potential targets and active compounds of XBF concerning the treatment of DPN.

**Results:**

The study successfully identified a total of 122 active compounds and 272 targets associated with XBF. 5 core targets of XBF for DPN were discovered by building PPI network. According to GO and KEGG pathway enrichment analysis, the mechanisms of XBF for DPN could be related to inflammation, immune regulation, and pivotal signalling pathways such as the TNF, TLR, CLR, and NOD-like receptor signalling pathways. These findings were further supported by immune infiltration analysis and localization of immune organs and cells. Moreover, the molecular docking simulations demonstrated a strong binding affinity between the active chemicals and the carefully selected targets.

**Conclusion:**

In summary, this study proposes a novel treatment model for XBF in DPN, and it also offers a new perspective for exploring the principles of traditional Chinese medicine (TCM) in the clinical management of DPN.

## 1 Introduction

Diabetic peripheral neuropathy (DPN) ranks among the most common complications of diabetes mellitus (DM). It refers to the aberration in sensory, motor, and autonomic functions of the peripheral nervous system, resulting from diffuse and focal neurological damage. The initial symptoms of DPN may comprise numbness, tingling or burning sensations, and severe pain, usually affecting the toes and symmetrically distributed (1). In the advanced stages of disease progression there are often numerous complications ranging from recurrent lower limb infections, ulcers and even amputations (2). Presently, the mechanism underlying DPN remains incompletely elucidated, with identified connections to neuronal inflammation, oxidative stress, and mitochondrial dysfunction. Aside from glycemic control, symptomatic relief stands as the sole recourse for the painful manifestations of DPN, yet falls short of an ideal solution, Opioids commonly used in clinical practice, such as tramadol and tapentadol, along with alternative opioid options, demonstrate short-term effectiveness in pain reduction for patients with diabetic peripheral neuropathy (DPN). Nevertheless, extended opioid use poses notable risks, including dependence, overdose, and potentially fatal consequences (3–5).

Traditional Chinese Medicine (TCM) encompasses enormous potential to prevent and treat complex and intractable illness including metabolic diseases, autoimmune diseases, and painful disorders (6, 7). The Xuebifang (XBF) Decoction is a combination of Astragali Radix (Huangqi in Chinese), Cinnamomi Ramulus (Guizhi in Chinese), Spatholobi Caulis (Jixueteng in Chinese), Paeoniae Radix Alba (Baishao in Chinese), Polygoni Multiflori Caulis (Shouwuteng in Chinese), and Glycyrrhizae Radix (Gancao in Chinese), which is derived from a classic TCM prescription Huangqi Guizhi Wuwu Decoction (HGWD). Numerous clinical studies have substantiated that HGWD can alleviate clinical symptoms and enhances neuronal function in DPN patients (8). In a prior investigation conducted by our research team, the co-administration of XBF and aconite demonstrated notable alleviation of severe neuropathic pain in DPN individuals, with no reported adverse events throughout the treatment duration. Simultaneously, a significant reduction in 2-hour blood glucose levels was observed in the patient cohort (9). As modern pharmacology advances, there is an ongoing exploration into the mechanism of action of XBF. Based on previous studies on HGWD and XBF, it’s suggested that XBF treatment may involve amelioration of neuronal function and blood glucose reduction, and the underlying mechanism is needed to be discovered.

**Figure 1:**
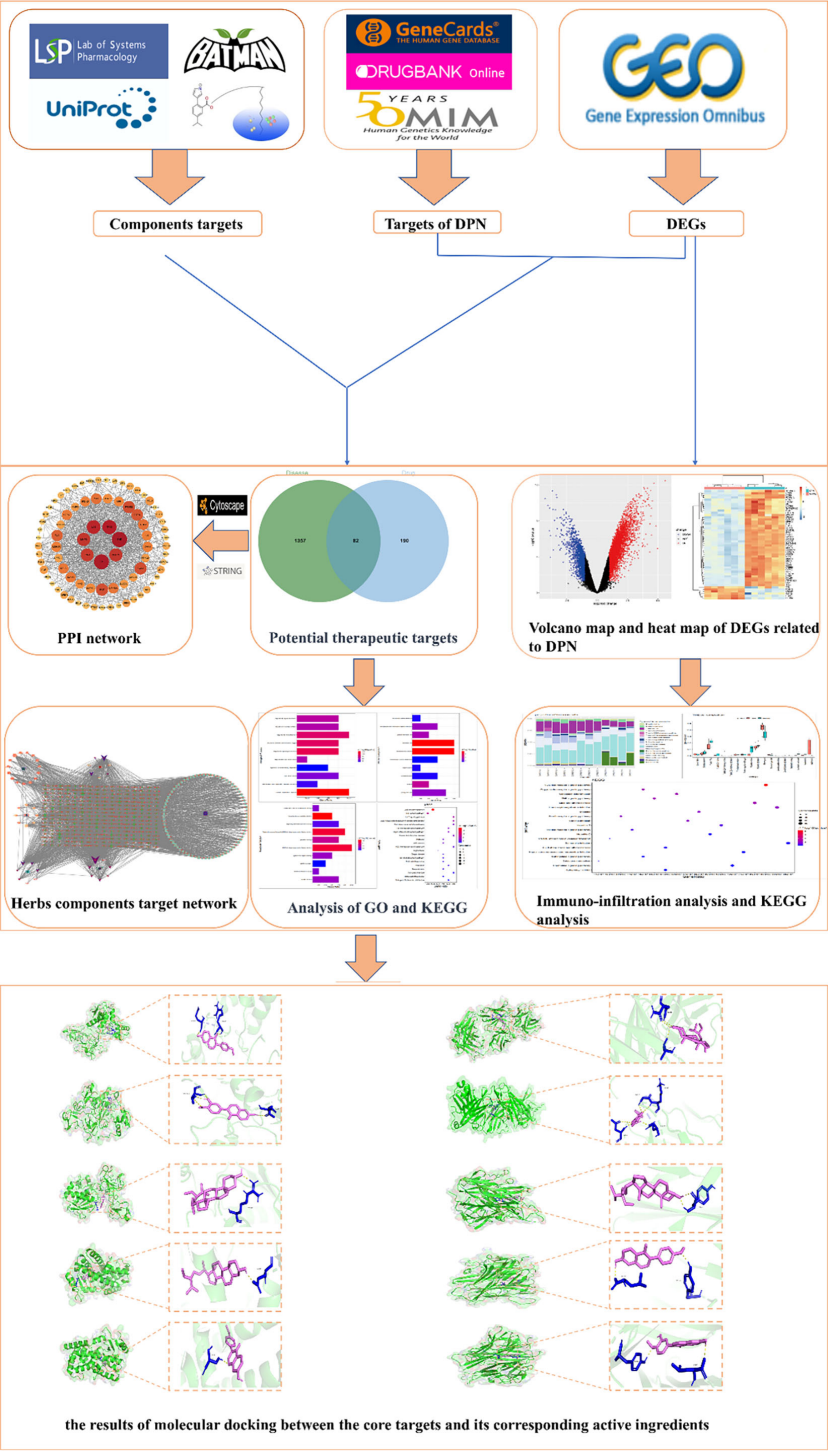
The workflow of the network pharmacological investigation strategy of XBF for DPN includes four parts: database preparation, network construction, GO and KEGG pathway analysis, and molecular docking validation.

Network pharmacology, as one of the most advanced research methods, has achieved remarkable progress in elucidating the principles of TCM prescriptions, identifying effective active chemical compounds, and pinpointing therapeutic targets of TCM (10, 11). With the recent convergence of bioinformatics, computational prediction-based network pharmacology has emerged as a powerful methodology to systematically unravel the biological mechanisms of complicated diseases and pharmaceutical effects from the molecular level (12). In this study, a combination of network pharmacology, bioinformatics, and molecular docking methods was employed to predict the primary bioactive chemicals, potential therapeutic targets, and signaling pathways of XBF in treating DPN. The aim is to offer a foundation and reference for understanding the potential therapeutic mechanism of XBF in managing DPN. The whole process of this study is shown in **Figure 1**.

## 2 Materials and methods

### 2.1 Screen the active ingredients and targets of XBF

The active compounds of BS, GC, HQ, GZ, and JXT were obtained from the TCMSP database(TCMSP: https://tcmsp-e.com), applying the criteria of Oral Bioavailability (OB) ≥ 30% and drug-like properties (DL) ≥ 0.18 (13). The active compounds of SWT were sourced from the BATMAN database (14). Subsequently, we utilized the TCMSP database and PharmMapper to identify the *in vivo* targets of the active compounds from Chinese herbal chemicals (15). The target names were mapped to gene names using the UniProt database (https://www.uniprot.org) (16). To explore the scientific and rational relationship between the effective chemicals in Chinese herbal medicine and their target genes, we constructed and visualized the TCM compound-target gene network using Cytoscape 3.9.1.

### 2.2 Collecting targets of DPN

Therapeutic targets were identified by querying the OMIM(https://omim.org.2020-11-14), Drugbank database (Drugbank, https://www.drugbank.ca/.2020-11-14) (17) and GeneCards platform (https://www.genecards.org/) (18) with “DPN” and “diabetic peripheral neuropathy” as keywords. The obtained results are subsequently converted into gene names using the UniProt database. Finally, we obtained the DPN target by removing duplicate genes.

### 2.3 Screen of DPN DEGs

To assess the mRNA expression of DPN and healthy individuals, we conducted an integrated query of gene expression data sets from the GEO database (www.ncbi.nlm.nih.gov/geo/). (GSE95849). We utilized the SVA and limma packages in R for batch correction and to identify differentially expressed genes (DEGs). The screening conditions for DEGs were p-value < 0.05 and |log2(foldchange)| > 1. Data visualization was accomplished using the ggplot2 and pheatmap packages in RStudio.

### 2.4 XBF acts on the DPN target

Disease targets retrieved from the Genecards, OMIM, and Drugbank databases were merged with DEGs obtained from the GEO database. Subsequently, this combined dataset underwent screening to identify targets present in at least two databases. These overlapping targets were then compared with the known XBF action targets to identify potential targets through which XBF may exert its therapeutic effects in the context of DPN.

### 2.5 Protein-protein interaction network of common targets

We uploaded a list of common targets for Homo sapiens to the STRING database and obtained a TSV file of protein-protein interactions, which we used to construct a network in Cytoscape 3.9.1[7]. To identify hub genes, we applied the cytoHubba plugin and calculated the degree centrality (DC), betweenness centrality (BC), and closeness centrality (CC) parameters. We employed three methods to determine the central gene: (1) identification of the top 10 genes with significant overlap among the three parameters; (2) cluster analysis using the MCODE plugin to generate highly connected sub-networks; and (3) filtering the core network using an average degree value greater than twice. By employing our approach, we can pinpoint key genes in the network that likely play a significant role when XBF interacts with the human body.

### 2.6 The analysis of GO and KEGG

In order to reveal the potential role of XBF and its impact on DPN, we conducted GO and KEGG enrichment analyses using R, and the results were visualized with a significance threshold set at *p ≤* 0.05. Furthermore, we performed KEGG enrichment analysis for DEGs using R language, and the outcomes were also visualized.

### 2.7 Molecular docking

Based on the herb-compound-target network, we identified 6 core components of XBF and further validated their potential therapeutic targets through molecular docking with 5 hub genes. The PubChem database was utilized to obtain the PubChem ID and 3D chemical structures, which were then converted to mol2 format using Open Babel (19). Protein crystal structures were obtained from the Protein Data Bank (PDB) (20), and were processed using PYMOL by dehydration, hydrogenation, and conversion to pdbqt format for molecular docking using Autodock tools software. The results of molecular docking were visualized using PYMOL, and the binding energies were utilized to screen the most promising chemical components of XBF for the treatment of DPN. In this screening process, we adhere to the principle that a reduced binding energy value signifies a more potent binding interaction between the ligand and receptor.

## 3 Results

### 3.1 Unraveling the bioactive constituents and putative molecular targets of XBF

**Figure 2:**
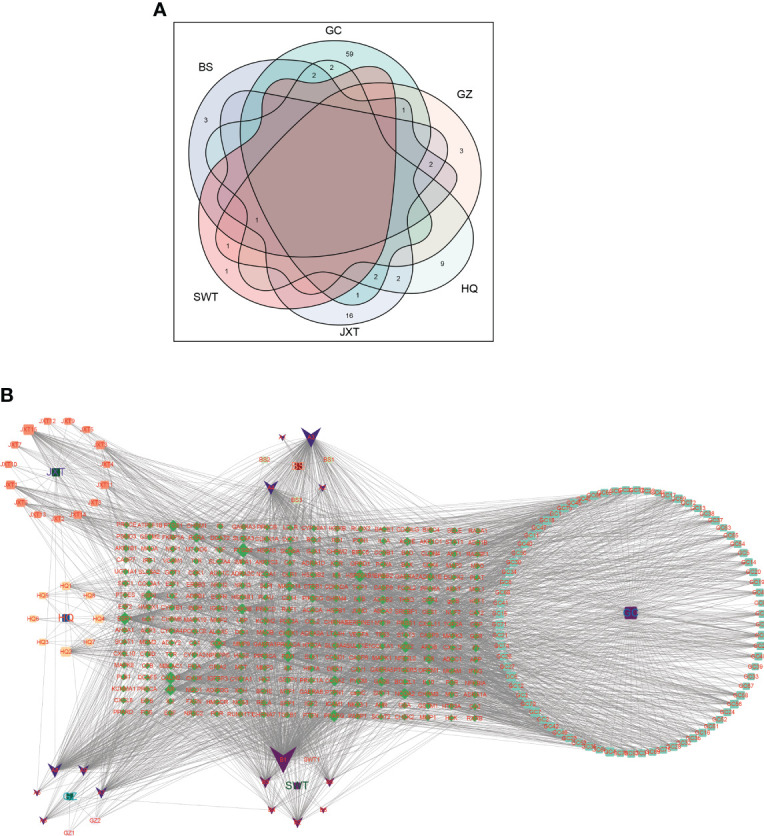
Screening of XBF active compounds and targets. **(A)** Venn diagrams showing numbers of active compounds in each herb of XBF. The Light cyan is HQ (17 compounds), Baby pink is GZ (6 compounds), cyan is GC (88 compounds), purple is BS (8 compounds), pink is SWT (2 compounds), heliotrope is JXT (23 compounds). **(B)** Herb–compound–target network. The network of the relationship between the active ingredients and the targets of XBF.

We obtained a total of 144 active chemicals of XBF from the TCMSP database and the BATMAN database, which yielded 122 active ingredients and 272 gene symbols of XBF after removing duplicates (**Supplementary Tables S1**, **S2**). Using a Venn diagram, we showed the distribution of active chemical components in each herb of XBF (**Figure 2A**). Subsequently, we utilized Cytoscape 3.9.1 software to construct a TCM compound-target gene network map, in which quercetin, kaempferol, beta-sitosterol, formononetin, isorhamnetin, and calycosin were identified as key nodes (**Figure 2B**).

**Figure 3:**
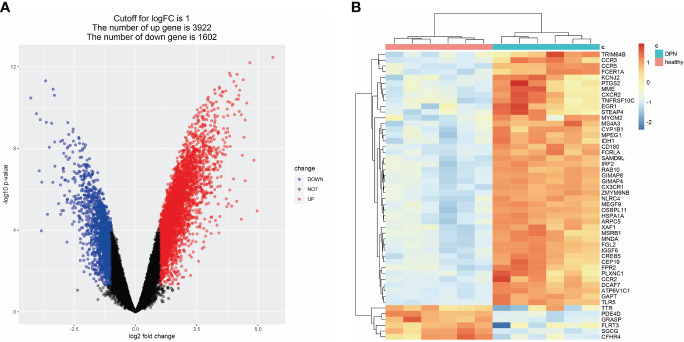
DEGs related to DPN in the GEO dataset. **(A)** Volcano map of DEGs related to DPN (GSE95849). **(B)** Heat map of DEGs related to DPN (GSE95849).

To identify DEGs associated with DPN, we compared gene expression levels between normal and DPN patient groups and identified 3922 up-regulated genes and 1602 down-regulated genes, which are presented in a heatmap of the top 50 DEGs (**Figures 3A, B**; **Supplementary Table S3**).

We collected 14 DPN-related targets from the Drugbank database, 348 related targets from the OMIM database, and 5195 related targets from GeneCards (**Supplementary Table S4**). To identify potential therapeutic targets for XBF, we intersected the DEGs with disease targets obtained from each of the three disease databases. We retained targets that appeared in at least two of the databases and overlaid them with the targets of XBF. By implementing this strategy, we identified a total of 81 putative targets (**Supplementary Table S4**).

### 3.2 PPI network

**Figure 4:**
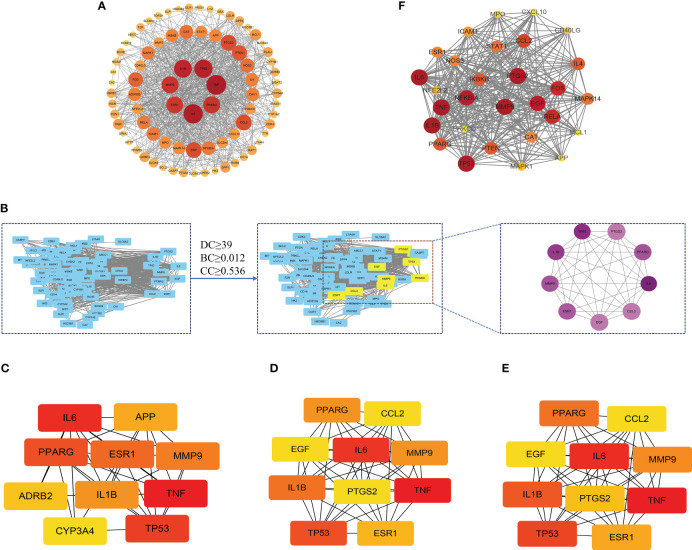
The protein–protein interaction (PPI) network of XBF’s targets for the treatment of DPN. **(A)** Analysis results of PPI network. **(B)** The process of topological screening for the PPI network. The 9 core targets was obtained by screening 81 common targets through DC, BC, CC. **(C–E)** The hub genes were selected from the PPI network using the CytoHubba plugin. The node color was from pale yellow to red, and the corresponding degree gradually larger. **(F)** PPI network based on clustery analysis using the MCODE plug-in.

In order to delve into the potential mode of action of XBF on DPN, we constructed a PPI network comprising 81 putative targets using the STING database. We analyzed the resulting TSV files with Cytoscape software and identified a network consisting of 81 nodes and 780 edges, with median values of 19.259, 0.536, and 0.012 for degree centrality (DC), closeness centrality (CC), and betweenness centrality (BC), respectively (**Figure 4A**). Through topological analysis, we filtered the core PPI network using a criterion of greater than 2 times the median DC, which yielded 9 core targets (**Figure 4B**). Additionally, we used the CytoHubba plugin of Cytoscape to identify the top 10 targets based on DC, BC, and CC separately, and identified the intersection of the three parameters as the core targets (**Figures 4C–E**). Finally, we utilized the MCODE plugin to perform clustering analysis and generate highly connected sub-networks (**Figure 4F**).

### 3.3 GO enrichment analysis

**Figure 5:**
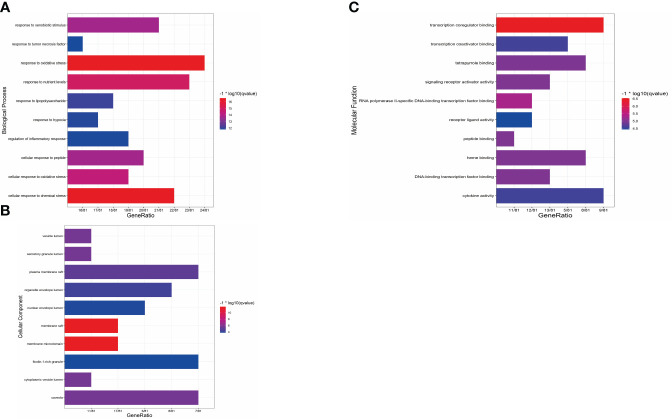
Results of GO enrichment analysis. **(A)** Bar chart of the biological process category terms from GO enrichment analysis. **(B)** Bar chart of the cellular component category terms from GO enrichment analysis. **(C)** Bar chart of the molecular function category terms from GO enrichment analysis.

To gain deeper insights into the action principle of XBF in treating DPN, we conducted GO enrichment analysis on the 81 cross targets. Our analysis revealed 1598 biological processes (BP), 107 molecular functions (MF), and 48 cellular components (CC) that were significantly enriched (pvalue ≤ 0.05) (**Supplementary Table S5**). To visualize the results, we created bar charts using R, highlighting the top 10 enriched BP terms, MF terms, and CC terms (**Figures 5A–C**). The most highly enriched BP terms included response to peptide, response to lipopolysaccharide, and response to extracellular stimulus. The most highly enriched MF terms were STAT family protein binding, insulin-like growth factor I binding, and nitric-oxide synthase binding. In terms of CC terms, the enriched categories were membrane raft and plasma membrane raft.

### 3.4 KEGG analysis

**Figure 6:**
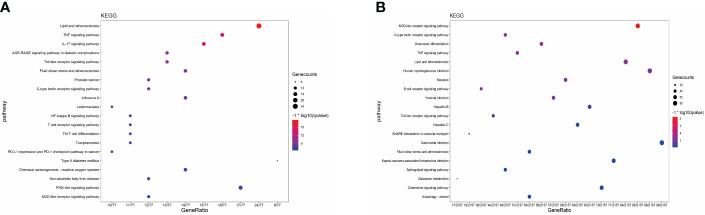
KEGG enrichment analysis. **(A)** KEGG enrichment analysis of XBF targets for DPN treatment. **(B)** KEGG enrichment analysis of DPN-related DEGs.

To gain deeper insights into the mode of action of XBF in treating DPN, we conducted KEGG pathway enrichment analysis on the 81 cross targets and DEGs. We found that both sets of targets were significantly enriched in similar pathways, such as TNF signalling pathway, Toll-like receptor (TLR) signalling pathway, C-type lectin receptor (CLR) signalling pathway, and NOD-like receptor signalling pathway (**Figures 6A, B**; **Supplementary Tables S6**, **S7**). These findings suggest that XBF may exert its therapeutic effects by targeting multiple pathways involved in inflammation and immune responses.

### 3.5 Immuno-infiltration analysis

**Figure 7:**
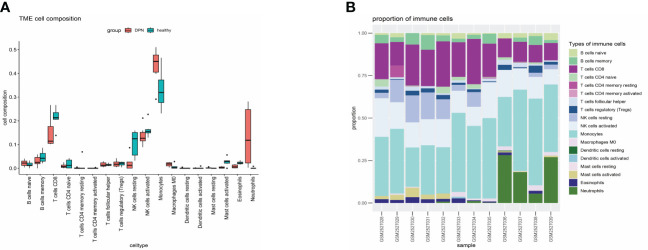
Immune infiltration analysis of the GSE95849 data set. **(A)** Boxplots. **(B)** Bar graph.

The previous KEGG analysis has revealed that the treatment of DPN with XBF involves an immune response. Therefore, we conducted an immune infiltration analysis of DEGs using R, based on the LM22 reference set and the CIBERSORT deconvolution algorithm with perm set to 1000. The results demonstrated that DPN patients had a higher proportion of Monocytes, Macrophages M0, and Neutrophils, and a lower fraction of B cell memory, T cell CD8, NK cells resting and Mast cells activated (**Figures 7A, B**). To obtain a more comprehensive insight into the precise localization of the potential therapeutic targets within immune cells and organs, we conducted a thorough analysis of their distribution via the online tool BioGPS (http://biogps.org/) (21). Our results revealed that out of the 81 putative targets of XBF, 34 exhibit diverse expression patterns across distinct immune cells and organs (**Table 1**).

**Table 1:** Distribution of immune cell/organ-specific expressed genes identified by BioGPS.

System/Organ	Genes	Count
Immune cells	CD14,KCNH2,TOP2A,CA2,CDK4,IL1B,CA1,TNF,STAT1,PRKCD PTGS1,DPP4,FOS,MCL1,ADRB2,PTGS2,CYP1B1,RXRA,PPKCD	19
Immune organs	MMP9,CD14,CAV1,TOP2A,SLPI,ADRB2,CA2,CA1,PTGS1,PTGS2 CYP1B1,FOS,NFKBIA,IL1B,PRKCD,MCL1	16

### 3.6 Molecular docking

**Figure 8:**
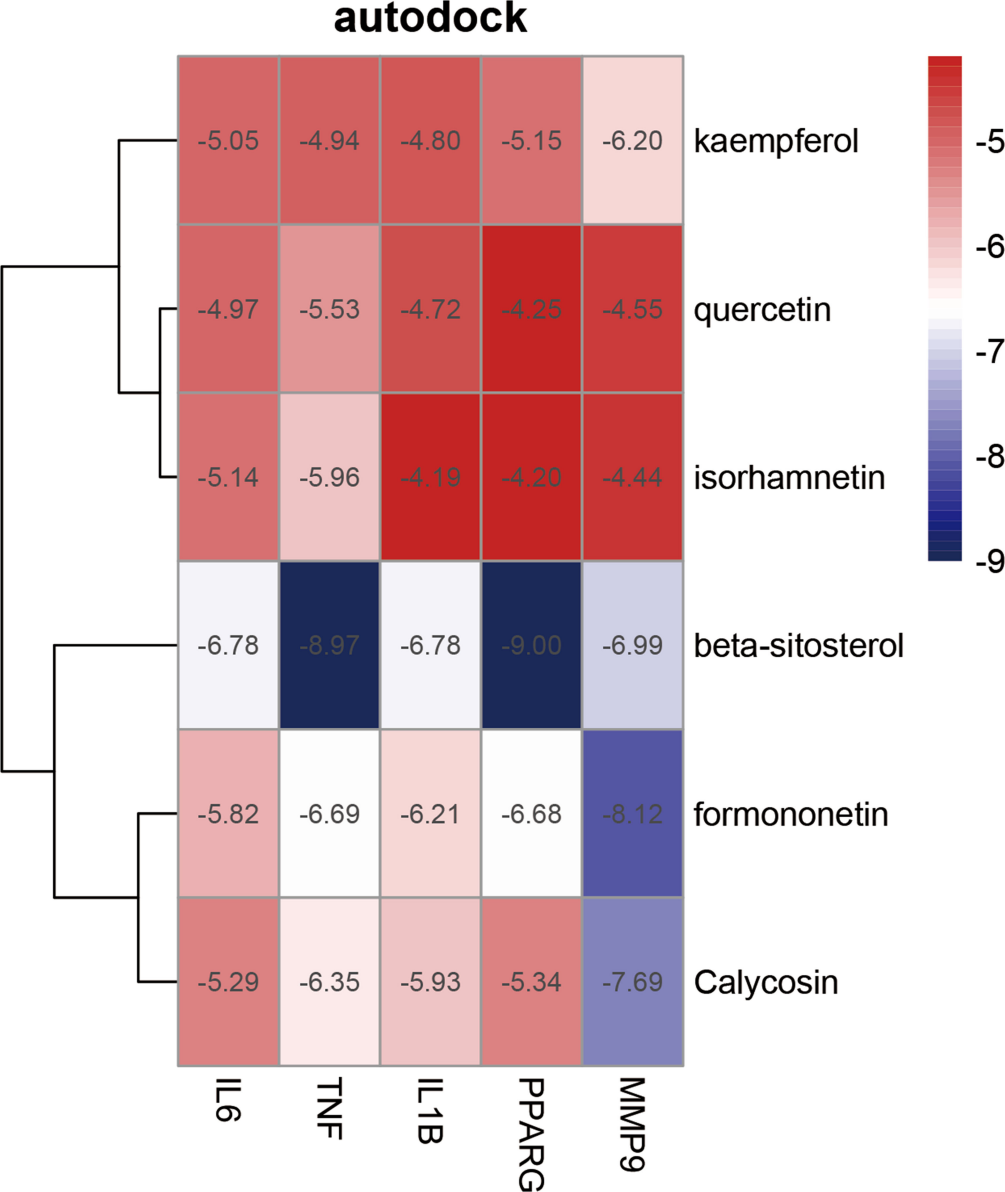
Heat map of molecular docking score. Binding energy(kcal/mol) of key targets and active compounds of herbs.

**Figure 9:**
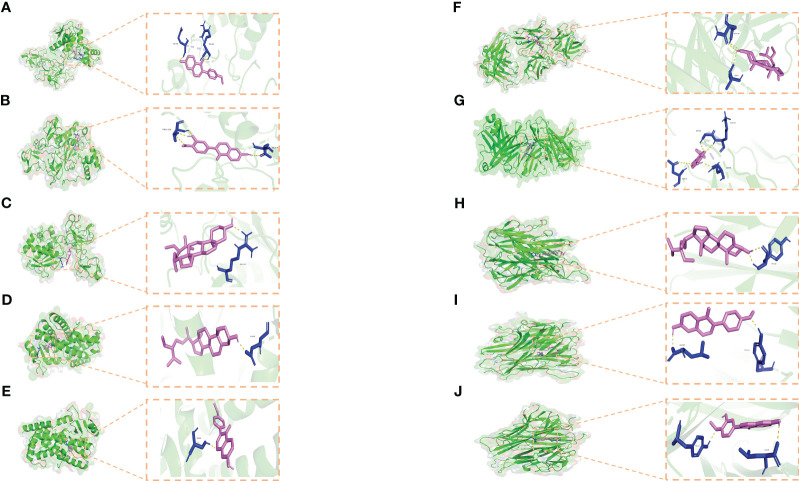
Molecular docking results. **(A–J)** Docking patterns of key targets and specific active compounds. Formononetin-MMP9 **(A)** Calycosin-MMP9 **(B)** β-sitosterol-MMP9 **(C)** β-sitosterol-PPARG **(D)** formononetin-PPARG **(E)** β-sitosterol-IL1B **(F)** formononetin-IL1B **(G)** β-sitosterol-TNF **(H)** formononetin-TNF **(I)** and Calycosin -TNF **(J)**.

To enhance the credibility of our network pharmacological analysis, we performed molecular docking simulations. These simulations allowed us to evaluate the interactions between the selected active compounds derived from Chinese herbal medicine and the core genes identified in the study. Our PPI network analysis identified 5 core targets, namely TNF, IL6, IL1B, MMP9, and PPARG. We also obtained the six core chemical components of XBF, including quercetin, kaempferol, beta-sitosterol, formononetin, isorhamnetin, and calycosin, through drug-ingredient-target network mapping. The core targets and components are first converted accordingly (**Table 2**). Molecular docking was then conducted as described in the methods section. The results of the 30 docking combinations were visualized in the form of a heat map and forms (**Table 3**; **Figure 8**), and the top ten with the lowest binding energy were shown in a separate figure (**Figures 9A–J**). Our molecular docking results indicate that the ligands bind tightly to the receptors, and the top three most tightly bound active ingredients were beta-sitosterol, formononetin, and calycosin.

**Table 2:** Details of targets and Compounds for molecular docking.

Molecule Name	PubChem ID	Target	PDB ID
quercetin	5280343	IL6	4NI7
kaempferol	5280863	TNF	7KP9
beta-sitosterol	222284	IL1B	5BVP
formononetin	5280378	PPARG	2HWQ
isorhamnetin	5281654	MMP9	1L6J
Calycosin	5280448		

**Table 3:** Binding energy of molecular docking.

	IL6	TNF	IL1B	PPARG	MMP9
quercetin	-4.97	-5.53	-4.72	-4.25	-4.55
kaempferol	-5.05	-4.94	-4.8	-5.15	-6.20
Beta-sitosterol	-6.78	-8.97	-6.78	-9.00	-6.99
formononetin	-5.82	-6.99	-6.21	-6.68	-8.12
isorhamnetin	-5.14	-5.96	-4.19	-4.20	-4.44
Calycosin	-5.29	-6.35	-5.93	-5.34	-7.69

## 4 Discussion

In our investigation, 122 active compounds of XBF and 272 associated target genes were sourced from TCMSP and BATMAN databases. We compiled 14 DPN-related targets from the Drugbank database, 348 related targets from OMIM, and 5195 related targets from GeneCards. Ultimately, we pinpointed 81 potential therapeutic targets for XBF. Through the construction of the PPI network, we identified five central target proteins: TNF, IL6, IL-1B, MMP9, and PPARG. Moreover, biological processes and pathways associated with inflammation and immune regulation were analyzed through GO enrichment, KEGG enrichment in conjunction with DEGs, including TNF signalling pathway, TLR signalling pathway, CLR signalling pathway, and NOD-like receptor signalling pathway.

The pivotal targets engaged by XBF in treating DPN encompass TNF, IL6, IL1B, MMP9, and PPARG. TNF-α, as a crucial immune cytokine, plays a significant role in the development and progression of various inflammatory, infectious, and autoimmune diseases (22). DPN leads to pathological changes in unmyelinated axons, causing axonal degeneration, and in myelinated nerve fibers, resulting in demyelination and diffused shrinkage. Notably, the expression of TNF-α can contribute to oligodendrocyte toxicity and demyelination in the context of DPN (23). TNF-α can induce the release of IL-1 and IL-6 from monocytes and endothelial cells (24). Indeed, the inhibition of IL-1β or IL-6 signalling has demonstrated efficacy in alleviating neuropathic pain caused by nerve injury, underscoring the crucial involvement of IL-1β and IL-6 in the pathogenesis of neuropathic pain (25). Recent investigations increasingly support IL-6 as a versatile cytokine with environment-dependent pro- and anti-inflammatory effects. It also plays a crucial role in stimulating remyelination and promoting axonal regeneration, signifying its diverse functions in neural repair and regeneration processes (26, 27). Concomitant expression of IL-1β in macrophages and isolated nerve fibers is also believed to be involved in maintaining the complex network of neural repair (28). Excessive MMP9 production results in the degradation or remodeling of the extracellular matrix, leading to compromised vascular supply to sensory nerve fibers, which, in turn, induces peripheral nerve dysfunction in individuals with diabetes (29). The downregulation of MMP9 has been observed to enhance peripheral nerve function by stimulating autophagy in Schwann cells, contributing to improvements in DPN (30). PPARs are a superfamily of nuclear hormone receptor ligand-activated transcription factors that serve a variety of physiological roles, among which the PPAR-γ subtype promotes glucose metabolism and insulin sensitization (31). Expressed in various tissues, notably nerves, PPAR-γ also shows antinociceptive abnormalities and antinociceptive allergic effects (32). It contributes to the metabolic homeostasis, while dyslipidemia mostly leads to atherosclerosis and reduced blood flow, which impairs nerve perfusion and further induces neurological dysfunction (33).

The targets of XBF in the treatment of DPN were distinctly enriched in pathways like the TNF, TLR, CLR, and NOD-like receptor signalling pathways. TNF-α signalling pathway may induce mechanically abnormal pain and thermal nociceptive sensitization through an autocrine mechanism by activating the NF-kB pathway, which is a major regulator of inflammatory response, insulin response and glucose metabolism (34, 35). Besides, TNF-α can trigger immune-mediated neuromyelin injury by sending signals to a range of different immune cell types, such as cDCs, NK cells, NKT cells, and T cells (36). Indeed, in animal models of diabetes, there is evidence supporting the association of the TLR signalling pathway and its downstream signalling molecules with diabetes-induced neuropathic pain, particularly during the early stages of the condition (37). Within the cell model, TLR induces the activation of MAPK and has a significant role in the regulation of neuronal plasticity (38, 39). CLR plays a crucial part in coordinating signalling pathways that induce regulation of adaptive immune responses (40). Signal transduction induced by the CLR signalling pathway appears to primarily activate or regulate NF-κB function, and its recognition of endogenous ligands serves a vital role in the homeostatic control of the immune system (41). Mincle is an activated CLR that senses damaged cells, and is also capable of recognizing glycolipid ligands on pathogens, implying that it may be linked to metabolic diseases such as diabetes (42). The innate and adaptive immune systems are primarily triggered by NOD-like receptor signalling pathways. These molecules can bind to the protein apoptosis-associated speck-like protein (ASC), leading to the stimulation of pro-IL-1 maturation and release as IL-1. Consequently, they play a role in mediating inflammatory responses (43).

Based on drug-ingredient-target network mapping and molecular docking, the top six pivotal chemical constituents of XBF were quercetin, kaempferol, β-sitosterol, formononetin, isorhamnetin, and calycosin. Among them, β-sitosterol binds most stably to PPARG. β-sitosterol, and a variety of medicinal plants, possesses antidiabetic, hypolipidemic, anticancer, anti-arthritic and anti-protective properties (44). β-sitosterol alleviates neuropathic pain through the inhibition of the TLR4/NF-κB signaling pathway, suppression of neuroinflammation, and modulation of microglial polarization towards an anti-inflammatory phenotype (45). Isorhamnetin markedly alleviates hypersensitivity to mechanical and thermal stimuli in nerve-injured rats by diminishing oxidative stress, restraining microglia and glial cell activation, suppressing the expression of inflammatory cytokines, and fostering M2 macrophage polarization (46). Isorhamnetin exhibits a multifaceted therapeutic impact on diabetes, encompassing anti-diabetic effects through insulin pathway regulation, modulation of carbohydrate metabolism, inhibition of NF-κB activation for vascular protection, and potential therapeutic benefits for diabetic neuropathy by alleviating neuropathic pain and improving nerve conduction velocity in rat models (47). In the acetic acid torsion test, formononetin markedly decreased the count of abdominal torsions, and its pain-alleviating effects were linked to a reduction in TNF-α, IL-6, and IL-1β levels. Additionally, it hindered neuronal apoptosis by activating the Nrf2/glutathione s-transferase pi 1 (GSTP1) signaling pathway and mitigated mechanical abnormalities in thermal injury sensitization and pain (48, 49). Calycosin is an isoflavone derived from Astragali Radix and contains antioxidant, anti-inflammatory, and immunomodulatory effects (50, 51). Calycosin accelerates diabetic wound healing in db/db mice by promoting the recruitment of anti-inflammatory monocytes and increasing the number of macrophages at the wound site, and also acts as a neuroprotective agent by reducing oxidative stress and delaying neuronal apoptosis (52, 53). Quercetin demonstrated significant efficacy in ameliorating neuropathic symptoms in diabetic rats. Its effects included improvements in nerve morphology, enhancement of paranodal junctions, down-regulation of key proteins, and restoration of gut microbiota balance through the modulation of specific microbial species associated with diabetic neuropathy (54). Meanwhile, quercetin demonstrates therapeutic promise in alleviating pain by inhibiting both peripheral and central sensitization. This effect is achieved through the modulation of the PAR2/TRPV1 signaling pathway and the suppression of inflammatory mediators via the toll-like receptor signaling pathway (55, 56). Kaempferol’s pain-relieving properties involve mitigating neuropathic pain and suppressing pro-inflammatory cytokines. This is achieved through the modulation of microglial polarization from the M1 to M2 phenotype, employing a mechanism that inhibits signaling pathways related to inflammation (57). Kaempferol has demonstrated the ability to rectify hyperglycemia, partially alleviate the pain response in diabetic rats, and modulate oxidative and nitrosative stress. Additionally, it reduces the formation of advanced glycosylation end products (AGEs) in diabetic rats, suggesting its potential application in the treatment of diabetic neuropathic pain (58).

In recent years, a growing amount of scientific research has emphasized the central role of neuroinflammation in the development of DPN (59). Inflammation stands as the central pathogenic mechanism and a primary therapeutic target for DPN (60). Low-grade intraneural inflammation could represent a shared converging pathway in the development of DPN. Factors such as iron deficiency, obesity, dyslipidemia, and rapid declines in HbA1c levels might contribute to neuropathy through inflammatory mechanisms (61). Persistent inflammation linked to diabetes plays a role in causing nerve damage, potentially worsening DPN. This impact extends to pain perception, involving heightened neuronal excitability, and influences the pain process by altering signaling in neurons (62). Sustained mild inflammation, initiated by the binding of glucose, lipoproteins, and oxidized proteins to neuronal receptors, plays a substantial role in the development of diabetic neuropathy. This process involves heightened immune cell activity, inflammatory signals, and disruptions in mitochondrial pathways (63). In T2DM, DPN entails the upregulation of inflammation-associated transcripts originating from macrophages within the dorsal root ganglia (DRG) (64). The contribution of TLRs, specifically TLR2 and TLR4, to the development of prediabetes and diabetic sensory neuropathy has been elucidated by mitigating the inflammatory response in both human subjects and various mouse models (65). Among the herbs in XBF, Astragali Radix demonstrates anti-inflammatory effects by downregulating the expression of inflammatory mediators, including iNOS, COX-2, IL-6, IL-1β, and TNF-α (66). Cinnamomi Ramulus extract alleviates neuroinflammation by attenuating the levels of pro-inflammatory cytokines, including IL-6, IL-1β, and TNF-α, in BV2 microglia (67). Polygoni Multiflori Caulis has demonstrated anti-inflammatory efficacy through the modulation of diverse inflammatory responses, including the realm of neuroinflammation (68, 69). The anti-neuroinflammatory properties of Paeonia lactiflora primarily involve inhibiting the production and release of inflammatory mediators, including cytokines, interleukins (IL-6, IL-1β), and tumor necrosis factor (TNF-α). This leads to a reduction in neuroinflammatory responses and associated injuries (70–72). Spatholobi Caulis effectively inhibits the release of pro-inflammatory mediators and cytokines (such as TNF-α, IL-1β, and IL-6) in inflammatory cells (73). The prevailing evidences suggests that a key mechanism of XBF in treating DPN involves the suppression of neuroinflammatory responses.

Alternatively, an expanding body of research highlights a notable link between DPN and immune regulation, with its pathogenesis intricately tied to autoimmunity (74, 75). Extensive infiltration of macrophages in DPN initiates neuroinflammation, culminating in neuropathy, pain, and sensory impairment. The aberrant involvement of macrophages in nerve repair has been investigated, uncovering that diabetes-induced macrophage recruitment and dysfunction may hinder effective nerve regeneration. Modulating macrophage function, particularly through nanomedicine and targeted drug delivery, holds promise for ameliorating DPN. This insight highlights immunomodulation as a promising avenue for advancing DPN treatment (76). In DPN, the activation of the receptor for hyperglycosylated end products (RAGE) instigates a pro-inflammatory response, primarily involving macrophages. Inflammatory macrophages contribute to the worsening of reduced insulin sensitivity, neuronal atrophy, and impaired nerve transport. Conversely, the absence of RAGE fosters anti-inflammatory macrophages, thereby safeguarding nerve function (77). This underscores the importance of immunomodulation in addressing diabetic neuropathic pain (78). Prominent lymphocyte infiltration, particularly CD3+ and CD8+ cells, in neural tissues of DPN patients, along with activated endoneuronal lymphocytes expressing cytokines and major histocompatibility class II antigens, indicates a potential involvement of immune cells in DPN pathogenesis through various effector mechanisms, including cytokine-mediated inflammation and microvascular inflammation (79). In a mouse model of DPN, persistent pain was observed despite sensory deficits, with late-stage pain correlating significantly with neutrophil and T-cell infiltration rather than ganglion cell stress markers, contrasting neuropathologic changes (80). The main component of XBF, Astragali Radix addresses immune-related conditions by modulating T-cell subsets (augmenting the quantity and function of CD4+ T-cells), boosting macrophage, natural killer cell, and lymphocyte activity, and regulating immune-related factors (such as IL-6, IL-10, IFN-γ, etc.) (81). 3-phenyl-propenal, extracted from Cinnamomi Ramulus, demonstrates immunomodulatory effects by suppressing TLR3, TLR4, and their downstream signalling components in Raw264.7 murine macrophages (82). Paeonia lactiflora, along with its principal active component paeoniflorin, demonstrates immunomodulatory effects by fine-tuning immune cell function, correcting disrupted signalling pathways, and maintaining a balanced distribution of immune cell subpopulations (83). This indicates that XBF may have therapeutic effects on DPN through the modulation of the immune system.

As with all scientific investigations, this study is not exempt from inherent limitations. Although cyberpharmacology endeavors to address the constraints associated with single-target investigations of herbal medicines and traditional Chinese medicine formulations, it encounters substantial challenges. Despite our utilization of dependable molecular docking techniques to validate the cyberpharmacology analysis results, we must acknowledge the following inherent limitations. Firstly, cyberpharmacology results critically hinge on the quality and precision of the bioinformatics and biomedical databases employed, introducing potential variability in data reliability. Secondly, cyberpharmacology often neglects the dose-response relationship of herbal components, overlooking the crucial aspect of Traditional Chinese Medicine (TCM) efficacy tied to achieving sufficient drug component levels at the target site. Thirdly, the differentiation of drug responses across diverse cell types and tissues may be inadequately addressed in network pharmacology, potentially leading to underestimation or overestimation of effects in actual biological systems. Fourthly, predictions in network pharmacology rely on established biological networks, yet for certain drugs or diseases, where the relevant mechanisms are not fully understood, predictive accuracy may be constrained. Fifthly, the predominantly static nature of network pharmacology approaches poses challenges in capturing the dynamic evolution of drug actions over time, a factor critical for certain diseases and treatment regimens. Sixthly, cyberpharmacology might overlook genetic and physiological individual differences, diverging from the core tenets of individualized medicine and the foundational philosophy of Chinese medicine emphasizing ‘diagnosis and treatment’ and ‘one person, one side’. Lastly, the intricate interactions inherent in multidrug combination therapy are challenging for network pharmacology to comprehensively consider, potentially limiting its practical applicability in treatment, particularly as Traditional Chinese Medicine compounds exert efficacy through the coordinated action of multiple components rather than a simple additive effect. Enhancing the predictive reliability involves subjecting XBF components to scrutiny through LC/MS analysis. The integration of artificial intelligence algorithms with network pharmacology is imperative for crafting dynamic models that comprehensively factor in cell and tissue specificity, consider drug dosage nuances, and incorporate environmental influences. Crucially, theoretical models must undergo validation via rigorous *in vivo* and *in vitro* experiments to bolster their credibility. Addressing these imperfections is pivotal for advancing the application of network pharmacology in Traditional Chinese Medicine (TCM) research.

## 5 Conclusion

In conclusion, we found that β-sitosterol, formononetin, and calycosin maybe the key active compounds of XBF that plays a therapeutic role in the treatment of DPN. Moreover, we revealed that the mechanisms of XBF are related to mitigating neuronal inflammatory responses and fostering the regulation of immune cells, mainly through targeting TNF, TLR, CLR, and NOD-like receptor signalling pathways.

## Data availability statement

The original contributions presented in the study are included in the article/**Supplementary Material**. Further inquiries can be directed to the corresponding authors.

## Author contributions

FH: Software, Visualization, Writing – original draft, Writing – review & editing. JL: Software, Visualization, Writing – original draft, Writing – review & editing. LX: Investigation, Validation, Resources, Writing – review & editing. ZL: Investigation, Validation, Writing – review & editing. WL: Methodology, Project administration, Resources, Supervision, Validation, Writing – review & editing. YZ: Funding acquisition, Methodology, Project administration, Resources, Supervision, Validation, Visualization, Writing – review & editing.
